# Editorial: Cochlear Implants and Music

**DOI:** 10.1177/23312165241231685

**Published:** 2024-02-15

**Authors:** Deborah A. Vickers, Brian C. J. Moore

**Affiliations:** 2152 University of Cambridge, Cambridge, UK

Music enjoyment is a holistic experience and is approached and experienced differently by different individuals. Engagement with music can happen in many different ways, such as listening to it via headphones or loudspeakers, attending a performance, playing a musical instrument, or dancing. In whatever ways music is enjoyed, it is a life-enriching experience that plays a role in the lives of many people.

People with cochlear implants (CIs) hear music differently from people with normal hearing (NH). Many post-lingually deaf adults who enjoyed music prior to implantation are unhappy with music listening with their CI. The two main reasons for this are: (1) melodic information carried in patterns of variation in fundamental frequency (heard as variations in pitch) is poorly conveyed by CIs; (2) it is hard to “hear out” the different instruments or voices in a mixture when listening via CIs.

The articles in this special issue cover a range of topics concerned with music listening via CIs. The articles are written by various professionals and patients with insight into music listening experiences. The special issue is largely based on presentations at the 3^rd^ International Music and CIs Conference, which was held in Cambridge, UK in 2021.

In current medical research practice, the opinions and experiences of the patient are considered as critical in informing relevant and meaningful research directions. In keeping with this practice, there are two articles in the special issue for which CI users were the researchers or co-creators of the article.

The article by Gfeller et al. reflects the musical listening and audiological practice experiences of adults using CIs. This article was co-authored with CI users. The basis for the research arose from a CI user panel at the 3^rd^ International Music and CIs Conference. The user panel was followed up with an open-ended questionnaire to gather further details about how music is or could be used in appointments where re-mapping (i.e., re-programming) of CIs is performed, and also to understand what CI users can do for themselves to maximise the benefit of listening to music. The findings showed that CI users were seldom offered the opportunity to set up their CIs to optimise music listening. Music outcomes were rarely considered within clinical appointments and very little music training was offered during rehabilitation. The authors developed recommendations that were compiled into the “Reciprocal Model for Music Rehabilitation” for use in clinical practice.

The article by Veltman et al. describes a music training programme (Musi-CI) designed to overcome the aversion that some post-lingually deaf adults have to listening to music with their CIs. The development of the programme was led by an adult musician (a CI user) who employed a multi-disciplinary consortium of advisors (professionals) to discuss and modify the programme using a participatory action research framework with 37 CI users. The resulting Musi-CI covers many musical attributes. Efficacy will be evaluated in future research.

Some of the articles in the special issue explore the perception of specific cues that underpin music-listening experiences.

Pathre and Marozeau examine the use of temporal cues for signalling emotion in music. Tempo has been suggested to be a prominent cue for emotional experience for CI users but there is some debate about how useful this cue is and what aspects convey emotion. The research was conducted with 12 NH listeners using pieces of music played at various tempos on conga drums to retain temporal cues but remove melodic cues, hence roughly simulating the listening experience of CI users. Each piece of music was rated on a scale going from happy to sad. The authors present evidence that there was not a strong relationship between the usual definition of tempo and the experienced emotion. They introduce a new measure, the Mean-Onset-to-Onset Difference (MOOD) of successive notes, that shows a much stronger relationship with perceived emotion. The authors are planning to assess whether the MOOD measure predicts the perceived emotion of music for CI users.

Harding et al. evaluate how emotion perception is affected by temporal and spectral changes in vocoder simulations with NH listeners. They used both noise vocoders and a sinewave vocoder. This work was based on previous research showing that tempo could convey emotional arousal (mellow, lively) and that spectral factors related to fundamental frequency and harmony convey valence (musical positiveness). The research team wanted to understand the role of other temporal properties and how adapting the spectral and temporal properties can affect the perception of emotion. Twenty-three NH listeners judged musical stimuli in terms of joy, fear, serenity, and sadness. The use of sinewave carriers, that better represent temporal information, led to higher emotional categorisation accuracy than the use of noise carriers. With noise carriers, there was a small benefit of improving the representation of spectral information via the use of more sharply filtered bands. Arousal scores were similar for vocoded and non-vocoded stimuli, but valence scores were far lower for vocoded stimuli. The authors conclude that it is likely to be beneficial to focus on improving the presentation of temporal rather than spectral cues to enhance emotion perception for CI users.

Seeberg et al. measure how the perception of certain musical features develop over the first three months post-switch-on for nine post-lingually deafened and recently implanted adults. They assessed the discrimination of four musical features (intensity, fundamental frequency, spectral shape, and rhythm) using both behavioural measures and using the mismatch negativity (MMN) brain response to oddball stimuli. The MMN amplitude increased in absolute value over time for fundamental frequency and spectral shape discrimination, suggesting improved discrimination skills over time, but behavioural discrimination performance did not change markedly over time. The MMN amplitude in response to changes in rhythm and intensity changes did not significantly change over the 3-month time period. The MMN amplitudes for a group of 13 experienced CI users were significantly larger and nearer to those for NH listeners for all measures, suggesting that discrimination abilities do improve with long-term listening experience. Fundamental frequency discrimination was typically poorer than normal for the CI users, but rhythm discrimination fell within the normal range. The results indicate that it could take many months of listening experience for perceptual abilities to the reach their asymptotic values.

Other articles investigate the perceptual abilities involved in perceiving music and how these abilities might be affected by a variety of factors.

Lam et al. evaluate how pre-implant hearing and music experiences are related to music perception and enjoyment after implantation, using questionnaires and a three-alternative forced-choice task that required detection of a change in a single note of a synthetic piano chord. In the changed chord either the second note was lowered or the third note was raised. Older participants with greater experience with their CI scored better than younger participants with less CI experience. The authors argued that the CI users did not “hear out” the individual notes in the chords; rather the task was probably performed using temporal interactions between notes, i.e., beats. The authors plan to explore this hypothesis further. A longer duration of hearing difficulty prior to getting CI(s) was associated with higher music enjoyment scores when the quality of music heard through the CI(s) was better.

Landsberger et al. explore how musical intervals are perceived by single-sided deaf listeners with a CI on one side. The participants were given the task of selecting pre-recorded vowels with different fundamental frequencies to represent the notes in the tune Happy Birthday. Participants were tested using each ear separately and with both ears. The accuracy with which the musical intervals were chosen was was lower using the CI than when using the “good” ear. When using both ears, musical interval estimation was dominated by the good ear.

Mo et al. assess the effect of frequency response manipulations on the sound quality of popular music by adjusting the energy in different bands of the audio signal. The stimuli were songs with accompanying instruments. Thirty one adult CI users made comparisons using the MUltiple Stimulus with Hidden Reference and Anchor for CI users (CI-MUSHRA) rating scale. Increasing the gain at low- and mid-range frequencies improved sound quality but changing the gain above 2 kHz had little effect. The musically trained participants were more sensitive to changes than those without musical training. The authors speculate that benefit of increasing the low-frequency gain arose from greater audibility of the lyrics, greater salience of the music beat, or both.

The final group of articles look at how the signal can be augmented or changed to improve music listening experiences for users of CIs.

Verma et al. explore the impact of using vibrotactile stimulation on timbre perception for NH listeners and CI users. Participants made similarity/dissimilarity judgements in a multi-dimensional scaling experiment using synthetic stimuli varying in attack time and amplitude modulation (AM) depth. The audio stimuli were presented together with tactile stimuli that also varied in attack time and AM depth. For both groups of listeners, the evidence indicates that the perception of timbre was influenced by the tactile stimuli, but the similarity judgements of the NH listeners were dominated by the auditory stimuli while CI users gave greater weight to the tactile stimuli. The authors suggest that future devices might employ vibrotactile stimulation to enhance cues to timbre for CI users.

Tahmasebi et al. investigate the potential benefit of modifying the sound coding strategy used in CIs to make music with lyrics and accompaniment more accessible to CI users. The modifications included reducing the number of bands used for stimulation, enhancing noise reduction so as to reduce the level of background instruments, and using greater back-end amplitude compression to optimize the electrical dynamic range and enhance the voice contrast in sung music. Preliminary results of perceptual evaluations show that reducing the number of bands for stimulation and optimizing the electric dynamic range significantly improves understanding of lyrics of the songs. Questionnaire responses showed that the back-end compressor significantly improves music enjoyment.

Moore discusses the difficulties associated with compressing the very wide range of sound levels occurring in music, especially live music, into the very small dynamic range of electric hearing. In most CIs, this amplitude compression is achieved in two stages, via “front-end” slow- or fast-acting amplitude compression in one or a few frequency bands followed by instantaneous amplitude compression (“mapping”) of the individual channels signals. He provides insight into potential ways to improve these stages so as to enhance music enjoyment for CI listeners, based on the lessons learnt from studies of amplitude compression in hearing aids. Moore recommends approaches for improving the analogue-to-digital converters used in CIs and optimising amplitude compression systems so as to improve the perception of intensity contrasts in music.

To conclude, this special issue brings together 11 articles on music perception and enjoyment by CI users. The articles cover a wide range of topics, including understanding the patient perspective, consideration of the abilities of CI users and limitations of their music listening experiences, consideration of the cues that are available to CI listeners, and approaches to improving the delivery of those cues, and pathways for signal processing modifications to improve the perception and enjoyment of music for users of CIs. It is our hope that these articles will provide inspiration and ideas that will lead to substantial improvements in music listening for users of CIs.

